# Do Expectant Mothers Exhibit Different Autonomic Responses to the Infant Cry Stimuli at Home Versus in the Laboratory?

**DOI:** 10.1002/dev.70032

**Published:** 2025-03-10

**Authors:** Shane Denherder, Dylan Neff, Bailey Speck, Joshua Marchant, Rose McLaughlin, K. Lee Raby, Sheila E. Crowell, Elisabeth Conradt

**Affiliations:** ^1^ Department of Psychology University of Utah Salt Lake City Utah USA; ^2^ Department of Psychiatry Duke University Durham North Carolina USA

**Keywords:** electrodermal activity, heart rate, infant cry, respiratory sinus arrhythmia, virtual laboratory visits

## Abstract

The COVID‐19 pandemic introduced challenges for keeping participants and research assistants safe during laboratory visits. One solution was administering research assessments in the participant's home via an online platform, despite limited evidence of whether online tasks have similar effects as laboratory contexts. The present study compares physiological responses to a virtual adaptation of an infant cry stimulus—which is commonly used to evoke and measure autonomic nervous system responses among pregnant individuals—to a traditional laboratory‐based cry task. Respiratory sinus arrhythmia (RSA), electrodermal activity (EDA), and heart rate (HR) were collected during infant cry presentation from 120 pregnant women in their third trimester. Half of the participants observed the infant cry stimulus in the laboratory before the pandemic, and the other half had the task delivered remotely using online teleconferencing technology in their homes. Results revealed that EDA increased and RSA decreased in response to the infant cry stimulus. HR did not significantly change from baseline to the infant cry stimulus. Importantly, whether the participants watched the infant cry stimulus at home versus in the laboratory did not affect their autonomic responses to the stimulus. These results demonstrate the ability of remote tasks to elicit an attachment‐relevant stress response in pregnant women for remote data collection.

## 1 Introduction

The COVID‐19 pandemic forced many researchers to adapt their laboratory‐based research protocols to an online data collection format, with little time to validate these new methods (Gao et al. 2022; Gunnar et al. 2021; Tabachnik et al. 2022). The pandemic necessitated a revolution in data collection with the use of ambulatory physiological monitoring devices and remote monitoring, potentially allowing for the inclusion of previously understudied populations through in‐home participation. However, transitioning to virtual data collection presents its own set of challenges. While all experimental tasks suffer a certain degree of loss of control when conducted in a home setting, some tasks may prove invalid due to this contextual shift. Building upon prior efforts to validate online tasks during the pandemic (e.g., Gunnar et al. 2021), this study aims to validate an at‐home version of a prenatal cry task by comparing autonomic responses to a similar cry stimulus conducted in the laboratory.

Experimental laboratory stressors are often used to elicit autonomic nervous system responses in participants. The sympathetic nervous system mobilizes resources in response to unexpected or threatening stimuli and can be observed in the activation of eccrine sweat glands (Christie 1981), which is measured through electrodermal activity (EDA). These glands typically exhibit increased activity during controlled, stress‐inducing situations, making EDA a marker of sympathetic reactivity to experimentally induced stress (Boucsein 2012; Nomikos et al. 1968). The parasympathetic nervous system can be similarly activated and measured via heart rate variability (HRV) across the respiratory cycle, called respiratory sinus arrhythmia (RSA). These cyclic increases and decreases in heart rate (HR) are regulated by the 10th cranial (vagus) nerve, which is controlled in part by the parasympathetic nervous system (Berntson et al. 1993). When responding to acute stress, a decrease in beat‐to‐beat fluctuations in HR is often observed, suggesting the reduction of parasympathetic regulatory influences on HR (Beauchaine and Thayer 2015).

RSA often exhibits an inverse relationship to HR and possibly with EDA in the context of stress regulation (Ablow et al. 2013). A decrease in RSA can reflect an adaptive response to challenge, indicating parasympathetic withdrawal via the vagus nerve, allowing the body to mobilize resources (Porges 2007). Conversely, sympathetic nervous system activation is often associated with increases in HR and EDA, indicating a heightened state of arousal and preparation for action. In the context of parenting, physiological responses such as RSA, EDA, and HR provide insight into the autonomic regulation of caregivers when faced with child‐related stressors (Ablow et al. 2013).

In a controlled laboratory setting, the sound of an infant crying is a mild stressor that elicits an autonomic nervous system stress response (Ablow et al. 2013; Groh and Roisman 2009). These responses may be shaped by individual differences in attachment history and parental sensitivity. For example, Ablow et al. (2013) reported that women with secure‐autonomous attachment states of mind displayed greater RSA decreases during an infant cry stimulus, indicating heightened emotional responsiveness and concern for the infant. In contrast, women with an insecure‐dismissing attachment state displayed large EDA increases, reflecting an increase in sympathetic nervous system activity corresponding to self‐reported anger and a sense of rejection from the stimuli. In addition, Groh and Roisman (2009) reported in a sample of nonexpectant/nonparent young adults (30 male and 30 female) between the ages of 18 and 25 that secure base script knowledge was negatively associated with electrodermal reactivity to audio recordings of infant cry vocalizations. Finally, while studying maternal sensitivity, Joosen et al. (2012) found that highly sensitive mothers showed stronger RSA decreases to hearing an infant cry compared to less sensitive mothers.

Laboratory versions of the infant cry task control for as much environmental variability as possible by using the same video, television, volume, chair, and seating position. Completing the task in the lab also mitigates distractions uniformly across a sample. However, at the onset of the pandemic, many data collection protocols were necessarily adapted to virtual modalities (Gao et al. 2022). Researchers lost the ability to control for consistent volume, viewing distance, and seating position, with the environment potentially introducing distractions from household activities and interactions. Indeed, these largely uncontrollable factors could impact physiological responses to stress, raising questions of efficacy for the infant cry task when administered at home. In response to the pandemic, researchers (DuPont et al. 2022; Gunnar et al. 2021; Kothgassner et al. 2021) have analyzed the Trier Social Stress Test (TSST; Kirschbaum et al. 1993) in online and laboratory settings and found similar cortisol, salivary alpha amylase, and cardiovascular responses to both stimuli. To our knowledge, the validity of a remotely delivered infant cry stimulus task has not been examined.

Conceptually, the infant cry stimulus should evoke emotions relevant to parental caregiving—an activity that is likely more salient and unrestricted in the home compared to other psychological phenomena of interest. Paradoxically, it could be argued that hearing an infant cry in the participant's home environment might be more ecologically valid than in a laboratory setting. Indeed, Baucom et al. (2018) analyzed cardiovascular reactivity during marital conflict in both home and laboratory settings, finding that HR reactivity to a conflict was greater in naturalistic home settings compared to the laboratory condition. Although this example differs from the present study in population and measure, it points to the complexities of the autonomic nervous system as it relates to interpersonal processes and context.

### 1.1 The Present Study

The goal of the present study was to compare the autonomic activity of individuals in their third trimester of pregnancy while observing an infant cry stimulus video in a laboratory context versus a home setting. We hypothesized that participants’ physiological reactivity to the infant cry task using measures of EDA, RSA, and HR would not significantly differ between the at‐home and laboratory versions. Specifically, EDA and HR should increase, and RSA should decrease with the presentation of an infant cry video, regardless of task setting.

## 2 Method

### 2.1 Participants

A total of 130 pregnant women were sampled from an ongoing longitudinal study (*N* = 223) assessing the intergenerational transmission of emotion dysregulation. Participants were recruited through obstetric clinics in a Mountain West region–based university healthcare system. Recruitment was limited to mothers aged 18–40 with a singleton pregnancy without serious complications who were planning to deliver within the university's healthcare system. Participants were oversampled for high and low levels of emotion dysregulation to achieve a uniform distribution within the sample. The university's institutional review board approved this study. Though data collection was ongoing at the time of analysis, the recruitment endpoint for this study was determined by the availability of relatively matched samples for both at‐home and laboratory participants at the time of preregistration in February 2022 (preregistration link: https://tinyurl.com/mv5t7rs7).

At the onset of the pandemic, our research team was forced to move data collection online, dividing the sample into 60 participants who completed laboratory visits. The remaining 70 completed the prenatal visit remotely using Zoom teleconferencing technology (Gao et al. 2022). Of the 130 participants, 10 had unusable physiological data due to equipment setup issues, eight of whom were from the at‐home group. Additional missing data from the at‐home group included six participants with missing RSA and HR data due to unreliable signal quality and four with unusable EDA data, primarily caused by excessive movement. To assess whether the data were missing completely at random (MCAR), Little's MCAR test was conducted. The test indicated that missing data patterns were not consistent with MCAR for EDA (*χ* ^2^(1, *N* = 130) = 4.25, *p* = 0.039) or electrocardiogram (ECG) measures (*χ* ^2^(2, *N* = 130) = 9.47, *p* = 0.009). This pattern aligns with expectations, given the challenges introduced by the shift to online data collection and the differing setup protocols between the two methods, as detailed below. Demographic information for participants with valid physiological data (*n* = 120) can be found in Table 1.

**TABLE 1:** Demographic characteristics and between‐groups significance of laboratory and at‐home groups.

Demographic variable	Laboratory group *N* (%)	At‐home group *N* (%)	At‐home/laboratory group *χ* ^2^ sig.
Group N (%)	58 (48.3%)	62 (51.7%)	
Maternal race and ethnicity			*p* = 0.140
White	48 (82.8%)	42 (67.7%)	
Hispanic or Latinx	7 (12.1%)	15 (24.2%	
More than one race	3 (5.2%)	8 (12.9%)	
Black/African American	2 (3.4%)	3 (4.8%)	
Asian	1 (1.7%)	3 (4.8%)	
Hawaiian/Pacific Islander	1 (1.7%)	1 (1.6%)	
Native American/Alaska Native	1 (0.9%)	0 (0.0%)	
Decline to respond	2 (3.4%)	3 (4.8%)	
Education			*p =* 0.889
4‐year college degree	21 (36.2%)	22 (35.5%)	
Technical school/some college/Assoc. degree	15 (25.9%)	19 (30.7%)	
Graduate degree	16 (27.6%)	16 (25.8%)	
High school diploma/GED	4 (6.9%)	3 (4.8%)	
Less than high school diploma	2 (3.4%)	2 (3.2%)	
Household income			*p =* 0.422
Less than $30,000	9 (15.5%)	8 (12.9%)	
$30,000 to $75,000	17 (29.3%)	27 (43.5%)	
$75,000 to $150,000	23 (39.7%)	18 (29.0%)	
Greater than $150,000	6 (10.3%)	7 (11.3%)	
Decline to respond	3 (5.2%)	2 (3.2%)	
Relationship status			*p* = 0.027^*^
Partnered or married	54 (93.1%)	49 (79.0%)	
Other	4 (6.9%)	13 (21.0%)	
Child birth order			*p* = 0.525
First pregnancy	17 (29.3%)	24 (38.7%)	
Second pregnancy	23 (39.7%)	21 (33.9%)	
Third pregnancy	11 (19.0%)	12 (19.4%)	
Fourth pregnancy	5 (8.6%)	5 (8.1%)	
Fifth pregnancy	2 (3.4%)	0 (0.0%)	
	Laboratory group M (SD)	At‐home group M (SD)	At‐home/laboratory group *t* sig.
Maternal age	29.86 (4.24)	29.42 (4.10)	*p* = 0.562
Gestational age (weeks)	36.72 (1.03)	36.68 (1.24)	*p* = 0.831
Difficulties in emotion regulation	73.48 (24.97)	79.52 (21.82)	*p* = 0.161
Body mass index	30.87(5.95)	31.31 (7.58)	*p* = 0.753
Achenbach anxiety scale	5.39 (3.59)	6.16 (3.14)	*p* = 0.216
Center for Epidemiological Studies‐Depression	17.66 (11.60)	14.06 (9.26)	*p* = 0.112
UCLA Life Stress Interview—chronic stress	2.23 (0.35)	2.13 (0.39)	*p* = 0.124

* *p* < 0.05.

^**^ *p* < 0.001.

### 2.2 Procedures

Following recruitment, participants completed online surveys to determine study eligibility and to obtain demographic data. Participants were then scheduled for the prenatal portion of the study during their third trimester of pregnancy. Participants then either visited the laboratory or completed the prenatal visit from home. The differences between protocols are described below.

#### 2.2.1 Laboratory Visit

After obtaining participants’ informed consent, a trained laboratory technician used an alcohol disinfectant pad to clean the areas immediately below the right clavicle, below the heart on the left side, and on the right side, aligning triangularly with the first two areas. After allowing the areas to dry, the technician then adhered three 7% chloride gel ECG spot electrodes to the skin in these locations. Four of the same ECG electrodes and a disinfectant pad were used to collect impedance cardiography data (configuration specified below). Next, two 0.5% chloride gel electrodermal electrodes were placed on the thenar and hypothenar eminences of the palm and wrapped with an elastic medical wrap to ensure stability.

Following the autonomic monitoring setup, participants completed a 10‐min baseline period of rest in a quiet, comfortable room where they were instructed not to sleep, use their phones, cross their legs, or chew gum. The baseline was followed by an abbreviated TSST (Kirschbaum et al. 1993), omitting the math section. Participants then completed surveys, functioning as a 10‐min recovery period following the TSST. Following this, research assistants then turned on a wall‐mounted 42‐inch (122 cm) television screen located 50 inches (127 cm) from where the participant was seated and instructed the participant to watch a 5‐min video. The video sequence began with a calming scene of an ocean beach with the sound of waves lapping (*seascape baseline*) for 1 min. This was followed by a 1‐min scene of a 9‐month‐old female baby playing with toys, the caregiver's voice occasionally talking to the baby (*play* segment). Then, a one‐min scene was played with the same baby crying in distress without the caregiver responding (*cry* segment). The last min of the video returned to the calming seascape scene (*seascape recovery*). Approximately 4 s of blank screen separated each video segment, with an additional 5 s at the beginning and end of the sequence. After the video sequence was completed, participants were debriefed and thanked for their participation. Given the present study's preregistered aims, only data from the *seascape* and *cry* segments were analyzed.

#### 2.2.2 At‐Home Visit

The at‐home visit protocol retained the same experimental elements with a few key differences. Rather than participants visiting the lab, research assistants delivered a bag to their home containing all necessary physiological monitoring equipment, a Microsoft Surface Pro computer for data acquisition, two tablets to conduct the Zoom call with multiple camera angles, and a Verizon Wireless Mi‐Fi hotspot to increase consistency in internet connection across participants in different households. Participants were given written instructions for mounting and setting up the tablets to connect to a scheduled Zoom meeting, where an experimenter and a trained lab technician guided them through setting up autonomic data collection hardware. Participants were instructed verbally and visually to use a provided alcohol disinfectant pad to clean “right below the right collar bone, on the left side of the body below the level of the heart, and on the right side of the body below the level of the heart.” After allowing the areas to dry, the technician explained which premarked 7% chloride gel ECG spot electrode was to be placed in each location. Next, two 0.5% chloride gel electrodermal electrodes were to be placed with “one on the fleshy part of the palm below the thumb, and the other below the small finger on the other fleshy part of the palm.” Participants were asked to wrap the EDA electrodes with an elastic medical wrap to ensure stability.

After setup, the visit followed the same sequence as the in‐person laboratory visit, except that an initial resting baseline of 20 min was used for the visit to allow the participant to recover from having set up their own physiological monitoring equipment. The same infant cry video was remotely initiated on the 13‐inch (38 cm) Surface Pro computer with the volume set to 100%. Notably, the Surface Pro tablet's proximity to the participant was not explicitly controlled and thus varied across participants (e.g., on the participant's lap or on the kitchen table).

### 2.3 Measures

#### 2.3.1 Physiological Measures

HR, RSA, and EDA data were collected throughout the visit using MindWare Technologies’ (MindWare Technologies Ltd., Gahanna, OH) BioLab data acquisition software (version 3.1) and wireless mobile devices sampled at 500 Hz.

#### 2.3.2 Skin Conductance Responses (SCRs)

EDA data were collected in 30‐s epochs via sensors placed on the thenar and hypothenar eminences on the palm of the participant's nondominant hand (Dawson et al. 2016). SCRs were defined as a momentary increase in skin conductance level greater than 0.05 µS. Data were cleaned by trained research assistants using MindWare EDA version 3.1 and verified by multi‐angle video recordings of the participants’ sessions. SCRs were removed using a spline interpolation when they occurred simultaneously with movement of the participants’ nondominant hand, with extraneous stimuli (e.g., dog barking), or when responses came in rapid succession without a recovery of at least 0.001 µS between responses.

#### 2.3.3 Cardiogram

Electrocardiogram data were collected using a lead II plus ground torso electrocardiogram configuration (Berntson et al. 2007). Data were analyzed in MindWare Technologies’ HRV editor (3.1) by trained research assistants. This program employs a fast Fourier transformation and Hamming window function (Harris 1978) on 60‐s epochs of data. For in‐person laboratory participants, a proxy respiration signal was obtained to validate the high‐frequency band of HRV via a four‐lead spot sensor impedance cardiogram (Qu et al. 1986), which was low‐pass filtered and detrended. We observed in a previous sample that some pregnant participants had a higher respiration rate than the general adult population. To ensure accurate measurement of HRV across the respiratory frequency (Shader et al. 2018), we expanded the typically recommended high‐frequency band from 0.12–0.42 (Task Force 1996) to 0.12–0.60 Hz. This expanded frequency band accommodates the highest respiration frequency that we have measured in this population (maximum 28.5 breaths per min) and does not introduce known influences from other autonomic regulatory systems. Data processing was overseen by a senior researcher; MindWare's software automatically identified R peaks within the QRS complex which were visually inspected and corrected if needed by trained research assistants. All ambiguous data and outliers were reviewed for accuracy by the study data manager. HR and RSA were examined using 60‐s epochs.

### 2.4 Analysis Plan

To test whether participants exhibited different autonomic responses to the infant cry stimuli at home versus in the laboratory, we conducted repeated‐measures analyses of variance (ANOVAs) comparing the *seascape baseline* and *cry* segments as the within‐subjects variable, and at‐home or laboratory group as the between‐subjects factor. For EDA, scores per 30‐s epoch (two per segment) were averaged together for a segment score, while HR and RSA were analyzed in 60‐s epochs.

## 3 Results

### 3.1 Preliminary Analysis

To identify potential covariates, we examined group differences across demographic variables using chi‐square tests for categorical variables and *t*‐tests for continuous variables (see Table 1). There was a significant difference between groups in relationship status (*p* = 0.027), such that the at‐home group reported lower rates of being partnered or married (79%) compared to the laboratory group (93%). However, relationship status was not significantly correlated with the autonomic difference scores (cry segment mean—seascape baseline mean) for EDA (*r* = 0.04, *p* = 0.661), RSA (*r* = 0.12, *p* = 0.202), or HR (*r* = −0.16, *p* = 0.097). Because relationship status was not significantly associated with autonomic responding, it was not included as a covariate from the main analysis due to a lack of causal justification (Wysocki et al. 2022).

### 3.2 Main Analysis

**FIGURE 1:**
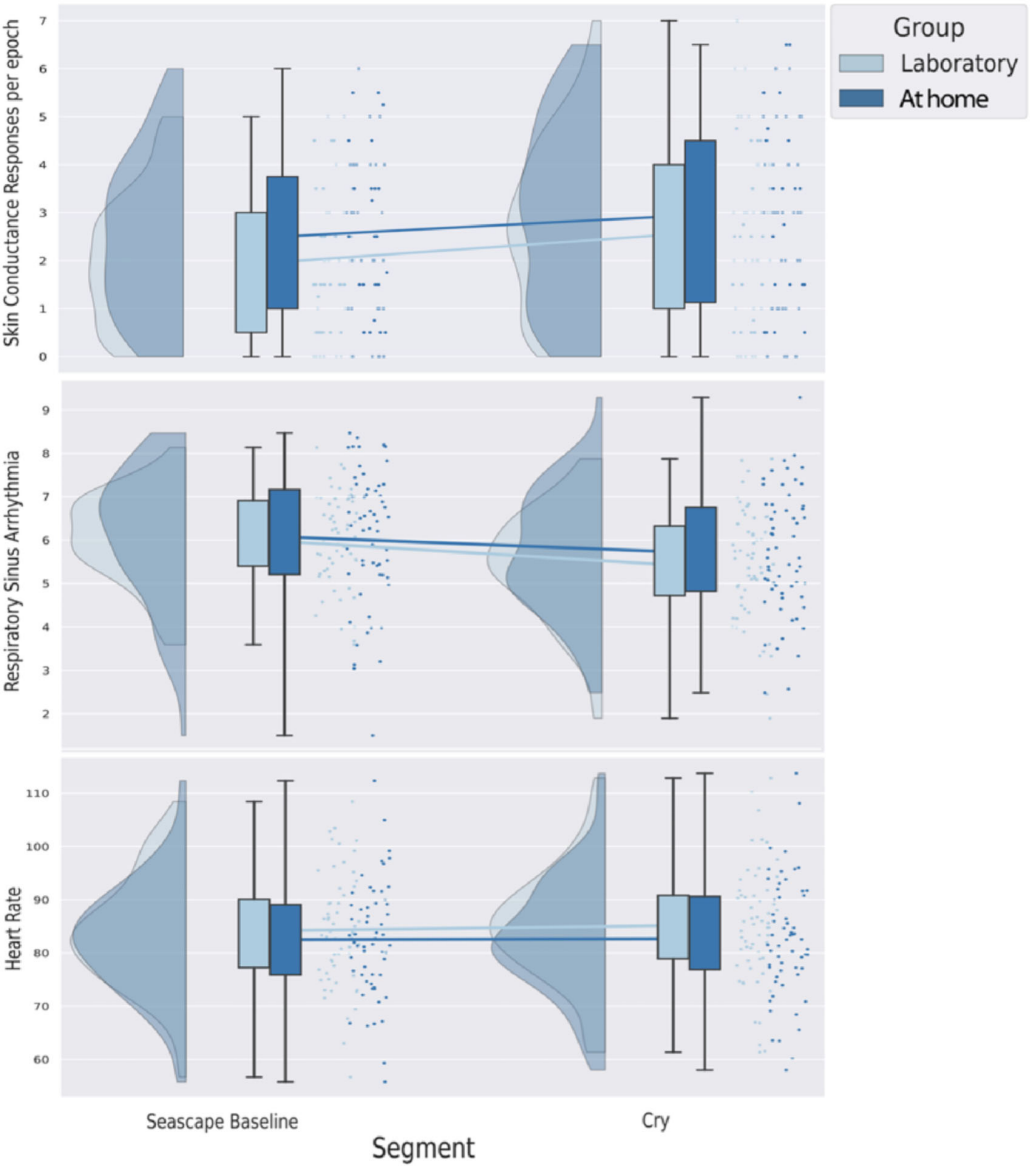
Raincloud (density, box and whisker, and scatter) plots for skin conductance response, respiratory sinus arrhythmia, and heart rate levels during the baseline and cry segments, separated by groups. Respiratory Sinus Arrhythmia, Heart Rate *n* = 114; Skin Conductance Responses *n* = 116.

We conducted three repeated‐measures ANOVAs to test whether the main effects of the infant cry stimulus on EDA, RSA, and HR differed for participants who viewed the stimulus in the lab versus at home. Between‐subjects effects, within‐subjects effects, and group interactions are reported in Table 2. Raincloud plots (density, box and whisker, and scatter plots) for RSA, SCRs, and HR are provided in Figure 1. As expected, given the nature of the task, there were significant main effects for EDA and RSA, such that EDA increased and RSA decreased from the seascape baseline to the cry segment. HR did not differ significantly between seascape baseline and cry segments. Importantly, there were no significant within‐subjects group interactions for SCRs, RSA, or HR. There were no significant mean differences between groups for SCRs, RSA, or HR.

**TABLE 2:** Descriptive statistics and within‐subjects analysis of variance of autonomic measures with main effects, group interactions, and between‐group differences of Laboratory and at‐home groups.

	Within‐Subjects Effects and Group Interactions						
Measure (DF)	Baseline		Cry		*F*	*p*	*η^2^*
	M	SD	M	SD			
SCRs (1, 114)	2.25	1.52	2.75	1.86	15.57**	< 0.001	0.120
SCRs × Group					0.425	0.516	0.004
RSA (1, 112)	6.09	1.26	5.53	1.32	40.36**	< 0.001	0.265
RSA × Group					0.054	0.816	< 0.001
HR (1, 112)	83.14	10.58	84.01	10.96	3.280	0.073	0.028
HR × Group					0.144	0.705	0.001
	**Between‐Groups Effects**						
Measure (DF)	Laboratory M		Online M		*F*	*p*	*η^2^*
	Baseline	Cry	Baseline	Cry			
SCRs (1, 114)	1.96	2.54	2.54	2.96	−3.05	0.083	0.026
RSA (1, 112)	6.01	5.43	6.17	5.63	0.608	0.437	0.005
HR (1, 112)	84.12	85.16	82.13	82.82	1.22	0.272	0.011

Abbreviations: HR = heart rate, RSA = respiratory sinus arrhythmia. SCRs = skin conductance responses.

^*^ *p* < 0.05.

^**^ *p* < 0.001.

## 4 Discussion

The present study evaluated whether pregnant women's autonomic responses to an at‐home infant cry stimulus differ from those observed in a laboratory setting. Consistent with prior research (Ablow et al. 2013; Groh and Roisman 2009; Joosen et al. 2012; Leerkes et al. 2015), pregnant participants showed significant RSA decreases and EDA increases in response to the infant cry stimuli. Importantly, autonomic responses to the cry stimulus video did not significantly differ in the laboratory and home settings. In other words, the at‐home cry stimulus appears to elicit a similar effect on participants’ parasympathetic and sympathetic nervous system function as the in‐laboratory cry stimulus.

Neither group displayed a significant change in HR from the seascape baseline to cry stimulus. This null result is consistent with Ablow et al. (2013), who also reported that women's HRs did not change significantly during a similarly structured cry stimulus task. However, Groh and Roisman (2009) found a significant change from baseline to cry stimulus when participants were first instructed to “try to think of how they would respond if the infant were their own child” (p. 890). While HR is often used as a physiological marker to measure stress response, it is important to point out that it is influenced by both the sympathetic and parasympathetic nervous systems, making its interpretation less straightforward than EDA (Ablow et al. 2013; Eagle et al. 2021). Importantly, a stress response can manifest with a rise in EDA (sympathetic activation) and a decrease in RSA (parasympathetic withdrawal), while HR might not show a significant increase because parasympathetic influences can modulate HR even as sympathetic arousal occurs. Still, there was no significant group interaction in HR reactivity to the infant cry stimulus in our sample. Thus, these findings offer support to the idea that observing the infant cry stimulus at home versus in the laboratory does not have differential effects on participants’ HRs.

### 4.1 Limitations

These findings are based on a sample of primarily well‐educated pregnant women whose modal annual household incomes were between $75,000 and $99,000. With the widespread adoption of work‐from‐home technologies (e.g., Zoom) at the start of the pandemic, this demographic group may have been more familiar with remote networking technologies and procedures and, therefore, more comfortable with the online visit setting than a more socioeconomically representative sample of pregnant people. Additionally, there were notable demographic differences between the laboratory and at‐home groups. The at‐home group reported lower rates of being partnered or married compared to the laboratory group. However, relationship status did not appear to affect autonomic reactivity in our sample. Still, the difference remains noteworthy and may reflect the increased accessibility of home‐based research studies for expectant parents without partner support.

Altogether, our findings indicate support for the validity of the at‐home infant cry stimulus. However, these results may not generalize to all samples of pregnant people. Future studies should aim to replicate the current results in more socioeconomically diverse samples. Lastly, it is essential to note that the present study collected data over two years beginning in September 2019. The laboratory group in this study conducted their visits before the COVID‐19 pandemic, while the at‐home group in this study participated during the pandemic. Although we found no differences in self‐reported stress scores between the two groups, nor differences in autonomic reactivity to the task, there may be complex stress regulation confounds in postpandemic participants for which our measures could not account.

## 5 Conclusions

The infant cry task elicits significant RSA and EDA responses in pregnant participants, and this response is not significantly different for those who completed the task in their homes despite the smaller size of the video device and the lack of experimental control over the environment. We believe these findings support the idea that the at‐home infant cry task is a valuable tool for developmental scientists studying maternal autonomic reactivity in the prenatal period. Importantly, validating online stress tasks such as the infant cry stimulus has the potential to overcome barriers to participation for pregnant individuals from underserved and underrepresented populations. Historically, data collection has presented challenges in reaching these populations. By utilizing online platforms, future developmental research can expand its samples to include individuals from more diverse backgrounds in terms of socioeconomic status and geographic location who may have otherwise been unable to participate in research conducted at large institutions.

## Author Contributions

**Shane Denherder**: conceptualization, formal analysis, writing – original draft. **Dylan Neff**: formal analysis, data curation, writing – review and editing. **Bailey Speck**: data curation, writing – review and editing. **Joshua Marchant**: methodology, writing – review and editing. **Rose McLaughlin**: writing – review and editing. **Lee Raby**: funding acquisition, formal analysis, supervision, writing – review and editing. **Sheila Crowell**: funding acquisition, writing – review and editing. **Elisabeth Conradt**: supervision and funding acquisition, conceptualization, formal analysis, writing – original draft, writing – review and editing.

## Conflicts of Interest

The authors declare no conflicts of interest.

## Data Availability

The data that support the findings of this study are available from the corresponding author upon reasonable request.
